# 

**Published:** 1871-02

**Authors:** 


					﻿Three Dollars a Year.
Single CoriEs, ;o Cents.
Vol. XXVIII.
FEBRUARY, 1871.
Number 2.
THE
(^^irago jJ^Fbiral journal.
J. ADAMS ALLEN. M.D., LL.D.. WALTER HAY, M.D.
IDI TORS.
CH ICAGO:
W. B. KEEN & COOKE, Publjshers,
113 & 115 State Street.
The postage on this ’Journal it Si cents a year—parable quarterly in advance.
NOTICE.— Messrs. William Baldwix A Co. No. 177 Browdrray. New York, are o«r
"-----* D—■ *—•»« for Tur Chjcaco Medical Jourxal
				

